# Disease stability and extended dosing under anti-VEGF treatment of exudative age-related macular degeneration (AMD) — a meta-analysis

**DOI:** 10.1007/s00417-020-05048-1

**Published:** 2021-02-02

**Authors:** Justus G. Garweg, Christin Gerhardt

**Affiliations:** 1 Swiss Eye Institute, Rotkreuz, and Berner Augenklinik am Lindenhofspital, Bern, Switzerland; 2 Department of Ophthalmology, Inselspital, University of Bern, Bern, Switzerland

**Keywords:** Age-related macular degeneration (AMD), Neovascular AMD, Treat-and-extend, Ranibizumab, Aflibercept, Brolucizumab

## Abstract

**Purpose:**

To assess disease stability (absence of intra- and/or subretinal fluid) and the portion of eyes being capable to extend their treatment interval to ≥ 12 weeks in exudative age-related macular degeneration (AMD).

**Methods:**

A systematic literature search was performed in NCBI, PubMed, CENTRAL, and ClinicalTrials.gov to identify clinical studies reporting treatment outcomes for ranibizumab, aflibercept, and brolucizumab in exudative AMD under a treat-and-extend protocol and a follow-up of ≥ 12 months. Weighted mean differences and subgroup comparisons were used to integrate the different studies.

**Results:**

This meta-analysis refers to 29 published series, including 27 independent samples and 5629 patients. In the pooled group, disease stability was reported in 62.9% and 56.0%, respectively, after 12 and 24 months of treatment, whereas treatment intervals were extended to ≥ 12 weeks in 37.7% and 42.6%, respectively. Ranibizumab, aflibercept, and brolucizumab differed regarding their potential to achieve disease stability (56.3%, 64.5%, and 71.5% after 12, and 50.0%, 52.7% and 75.7% after 24 months; *p* = < 0.001) and to allow an interval extension to ≥ 12 weeks (28.6%, 34.2%, and 53.3% after 12, and 34.2%, 47.7%, and 41.7% after 24 months; *p* = < 0.001).

**Conclusion:**

The portion of eyes achieving disease stability regressed in the second year, whereas the portion of eyes under a ≥ 12-week interval increased. This discrepancy may reflect the challenges in balancing between under-treatment and a reduced treatment burden.

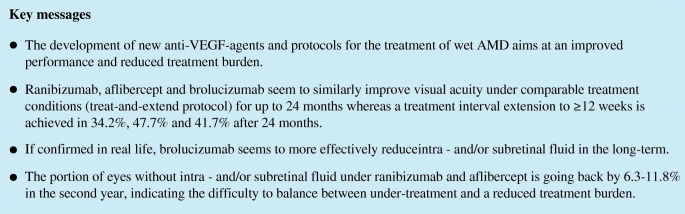



## Introduction

The introduction of intravitreal anti-vascular endothelial growth factor (anti-VEGF) drugs at the beginning of this century has revolutionized the treatment of exudative maculopathies [1]. Ranibizumab (Ran; Lucentis®, Genentech, South San Francisco, CA, USA) was the first intravitreal anti-VEGF treatment receiving FDA approval for the treatment of neovascular age-related macular degeneration (nAMD) in 2006 [2, 3]. By the time of its introduction, bevacizumab (Avastin®, Genentech/Roche, Switzerland), approved for the systemic use in oncology, had been broadly and successfully used as a low-cost off-label intravitreal treatment alternative for macular edema of different pathophysiologies [4] showing a success rate similar to Ran [5], but not necessarily a comparably favorable safety profile [6–8]. Therefore, its reimbursement by health insurance systems was not granted in many countries. Early clinical research focused on reducing the treatment burden due to monthly visits and injections according to the label, and on improving patient adherence and functional outcomes [9–11] as well as the performance of the overcrowded outpatient clinics [12]. Soon, it became evident that an impressive short-term effect was lost after 2 years once the patients were switched to an as-needed or pro re nata (PRN) therapy [13, 14] that could hardly be explained by the progression of the underlying atrophying macular situation [15, 16]. Based on the less enthusiastic real-life experience with long-term outcomes, research switched its focus on predisposing factors, treatment adherence, and treatment strategy modification [17–24]. When patients adhered to their treatment protocols, one-third still maintained a reading and driving vision over many years [25]. With aflibercept (Afl; Eylea®, Bayer, Berlin, Germany), a second treatment option became approved in late 2012 for the treatment of nAMD [26, 27], providing a new hope to overcome the limitations of long-term treatment. Since then, the discussion focused on the differences between the two drugs regarding their response in distinct lesion types, and the effect of switching treatment. A few months ago, the third anti-VEGF agent named brolucizumab (Bro; Beovu®, Novartis Inc., Basel, Switzerland) had been launched. The two approval studies (Harrier and Hawk [28, 29]) reported a more substantial effect on the reduction of retinal fluid and longer mean re-treatment intervals, promising less burden for patients and caregivers.

Whereas similar visual gains have been reported for the three drugs, a direct head-to-head comparison of their potential to dry the retina and enable extension of the treatment interval to 12 or more weeks was not available. This drove us to undertake a meta-analysis based on the published evidence. We investigated the potential of Ran, Afl, and Bro to achieve the absence of (intra- and/or sub-) retinal fluid and treatment intervals of ≥ 12 weeks based on comparable treatment conditions.

## Material and methods

A systematic literature search was performed on May 14, 2020, in the NCBI/PubMed database from the National Institute of Health, USA (https://www.ncbi.nlm.nih.gov/pubmed), as well as in the Cochrane Central Register of Controlled Trials (CENTRAL) and on ClinicalTrials.gov, to identify pro- and retrospective studies retrieved by the key terms ((age-related macular degeneration OR wAMD OR exudative AMD) AND (treat and extend OR T&E OR PRN OR pro re nata)). Furthermore, researchers in the field have been contacted for additional studies, and reference lists of meta-analyses and reviews have been screened for suitable articles. From the resulting set, all manuscripts published since 2008 were selected according to the inclusion criteria described below. Cross-references identified during a manual search of references from the retrieved articles were included if they provided additional data. Furthermore, several articles published on the same study could be included if they added additional information. However, only full-length articles in English were included. In contrast, articles reporting identical data from the same study, abstracts, letters to the editor, case reports, and review articles were excluded.

### Inclusion and exclusion criteria

Criteria applied for studies to be considered eligible for this meta-analysis were:

1. Study design: prospective and retrospective clinical studies and case series with ≥ 40 participants in total published or accepted between 2008 and 2020;
2. Population: treatment-naïve participants with exudative age-related macular degeneration (AMD) and a follow-up of at least 12 months;
3. Intervention: the following treatment schedules were accepted:
  Treat-and-extend (T&E) protocol starting with three monthly loading injections (in case of disease stability, the treatment interval was extended by 2 to 4 weeks to a maximum of 16 weeks; in case of instability, the interval was shortened by 2 to 4 weeks)
  Fixed treatment schedule (monthly or bi-monthly) during the first year followed by T&E protocol in the second year or capped PRN with mandatory treatment at least q12 weeks (in this case, only year 2 data were included)

For this study, a capped PRN protocol was accepted as used in the second year of the View studies and a primary extension to 12 weeks with the option to reduce the treatment interval to 8 weeks as in Harrier and Hawk trials as comparable to a T&E protocol.

The following outcome variables were defined:

- Proportion of eyes achieving disease stability (operationalized by the absence of intra- and/or subretinal fluid);

b. Proportion of eyes reaching dosing intervals of ≥ 12 weeks;
c. Best-corrected visual acuity and visual gain;
d. The number of injections and treatment retention rate.

### Information retrieved from the included studies

Whenever information was missing in the published papers, we consulted ClinicalTrials.gov or other study registries for further information. We extracted the following information: identifier, name of the study, type of anti-VEGF-agent, subgroups or treatment arms, treatment regimen and interval, study design, individual study definition for stability of disease, demographics (age, gender, country), follow-up period, values for visual acuity, number of injections, indices for stability (macular fluid, treatment interval extension), and retention rate for 12- and 24-month follow-up. Wherever necessary, we converted visual acuity scores into the Early Treatment Diabetic Retinopathy Study (ETDRS) letters to ensure consistency. As mentioned above, only treatment arms meeting T&E criteria or a fixed regimen followed by T&E or capped PRN criteria were included. In contrast, exclusively fixed dosing and regular PRN groups were excluded. Each included treatment arm is represented in one line in Table 1. To calculate a meta-analysis on these data, we extracted both mean and standard deviation whenever possible. In cases where the standard deviation was missing, we calculated the weighted mean of the whole sample. We replaced the missing value by this estimation to avoid a loss of data.

**Table 1:** Overview of included studies (in alphabetical order)

Study identifier	Study name	Anti-VEGF agent	Subgroup	Treatment protocol	Design	Sample size	Age mean	Age SD	Gender (% female)	Country	Duration of study (years)
Barthelmes 2018 [31]		Aflibercept 2.0 mg		T&E	Database observational study	212	78.4	7.6	62.0	Australia/Switzerland	2
Berg 2015, 2016 [41, 42]	LUCAS	Ranibizumab 0.5 mg		T&E	Multicenter, randomized, double-blind, non-inferiority trial	218	78.0	8.2	64.2	Norway	2
Cui 2018 [50]		Ranibizumab 0.5 mg		T&E	Retrospective case-controlled non-inferiority multiple center study	85	69.4	6.8	24.7	China	1
DeCroos 2017 [58]	ATLAS	Aflibercept 2.0 mg		T&E	Multicenter, prospective, open label, non-comparative, interventional study	40	81.3	7.4	57.5	USA	2
Dugel 2019, 2020 [27, 28]	HAWK	Brolucizumab 6.0 mg	HAWK q12w	T&E	Double-masked, multicenter, active-controlled randomized phase 3 trial	360	76.7	9.0	56.9	Multinational	2
Dugel 2019, 2020 [27, 28]	HARRIER	Brolucizumab 6.0 mg	HARRIER q12w	T&E	Double-masked, multicenter, active-controlled randomized phase 3 trial	370	74.8	8.6	56.8	Multinational	2
Eleftheriadou 2018 [55]		Aflibercept 2.0 mg		Bimonthly, then T&E	Retrospective, single-center, non-randomized interventional case series	148	80.6	8.3	58.9	UK	3
Gillies 2019a, b [36, 37], Novartis 2018 [38]	RIVAL	Ranibizumab 0.5 mg		T&E	Phase IV randomized, partially-masked, multicenter study	141	76.6	8.5	50.7	Australia	2
Gillies 2019a, b [36, 37], Novartis 2018 [38]	RIVAL	Aflibercept 2.0 mg		T&E	Phase IV randomized, partially-masked, multicenter study	137	78.7	7.5	54.7	Australia	2
Guymer 2019 [39]	FLUID	Ranibizumab 0.5 mg	intensive arm	T&E	Multicenter, randomized, 24-month, phase 4, single-masked, non-inferiority clinical trial	172	79.3	8.1	57.8	Australia	2
Guymer 2019 [39]	FLUID	Ranibizumab 0.5 mg	relaxed arm	T&E	Multicenter, randomized, 24-month, phase 4, single-masked, non-inferiority clinical trial	173	78.8	8.2	51.1	Australia	2
Jørstad 2017 [43]		Aflibercept 2.0 mg		T&E	2-year prospective study	50				Norway	2
Kertes 2019, 2020 [47, 48], Novartis 2018 [49]	CANTREAT	Ranibizumab 0.5 mg	T&E	T&E	Prospective, randomized, open-label, multicenter, non-inferiority, post-authorization study	287	78.9	7.7	60.6	Canada	2
Khurana 2019 [32], Schmidt-Erfurth 2014 [33]	VIEW	Ranibizumab 0.5 mg		Rq4 first year, then capped PRN	Double-masked, multicenter, parallel-group, active-controlled, randomized trials	595	75.6	8.7	57.3	Multinational	2
Khurana 2019 [32], Schmidt-Erfurth 2014 [33]	VIEW	Aflibercept 2.0 mg	2q4	2q4; then capped PRN	Double-masked, multicenter, parallel-group, active-controlled, randomized trials	613	75.9	8.2	60.4	Multinational	2
Khurana 2019 [32], Schmidt-Erfurth 2014 [33]	VIEW	Aflibercept 2.0 mg	2q8	2q8; then capped PRN	Double-masked, multicenter, parallel-group, active-controlled, randomized trials	607	75.8	8.5	58.1	Multinational	2
Mitchell 2020 [35]	ARIES	Aflibercept 2.0 mg	early start	T&E	Multicenter, randomized phase 4 study	135	76.0	8.8	60.0	Multinational	2
Mitchell 2020 [35]	ARIES	Aflibercept 2.0 mg	late start	T&E	Multicenter, randomized phase 4 study	136	76.9	8.2	53.7	Multinational	2
Ohji 2018, 2020 [51, 52], Wai 2018 [53], Bayer 2020 [54]	ALTAIR	Aflibercept 2.0 mg	4 W-Adj	T&E	Randomized, open-label, multicenter, Phase 4 study	123	74.0		27.6	Japan	2
Ohji 2018, 2020 [51, 52], Wai 2018 [53], Bayer 2020 [54]	ALTAIR	Aflibercept 2.0 mg	2 W-Adj	T&E	Randomized, open-label, multicenter, Phase 4 study	123	74.0		27.6	Japan	2
Prünte 2019 [44], Ebneter 2019 [45]	ASTERIA	Aflibercept 2.0 mg	EFF1Y	T&E	Retrospective and prospective, multicenter observational study	139				Switzerland	1
Prünte 2019 [44], Ebneter 2019 [45]	ASTERIA	Aflibercept 2.0 mg	EFF2Y	T&E	Retrospective and prospective, multicenter observational study	95	80.9	8.3	55.6	Switzerland	2
Silva 2018 [34]	TREND	Ranibizumab 0.5 mg	T&E	T&E	Masked, multicenter, randomized, interventional study	323	75.3	8.6	55.4	Multinational	1
Taipale 2020 [59]		Aflibercept 2.0 mg	T&E	T&E	Masked, multicenter, randomized, interventional study	26	76.6	6.9	65.0	Finland	1
Toalster 2013 [40]		Ranibizumab 0.5 mg	T&E	T&E	Prospective, multicenter, non-randomized trial	45	81.7	6.3	64.0	Australia	1
Traine 2019 [46]		Aflibercept 2.0 mg	T&E	T&E	Single-center retrospective observational study	231	79.9	8.2	62.3	Switzerland	4
Wykoff 2015, 2017 [56, 57]	TREX-AMD	Ranibizumab 0.5 mg	T&E	T&E	Phase IIIb, multicenter, randomized, controlled clinical trial	40	77.0		63.3	USA	2

*M*, mean; *SD*, standard deviation

### Statistical analysis

For a demographic overview of our studies, we calculated weighted mean values for the demographics age and sex ratio and frequency distributions for the country of origin. For the meta-analytic integration of results, we calculated weighted mean differences using random-effects models (Comprehensive Meta-Analysis (CMA) [30]) for visual acuity scores at baseline and 12- and 24-month follow-up, and for the number of injections and visual gain at 12- and 24-months follow-up. Patient retention and stability criteria were analyzed using subgroup comparison. All scores were calculated across all included studies as well as on subgroup level to compare anti-VEGF-agents (Ran, Afl, Bro). Subgroup comparisons were performed using *χ*^2^ test.

## Results

### Included studies

**Fig. 1:**
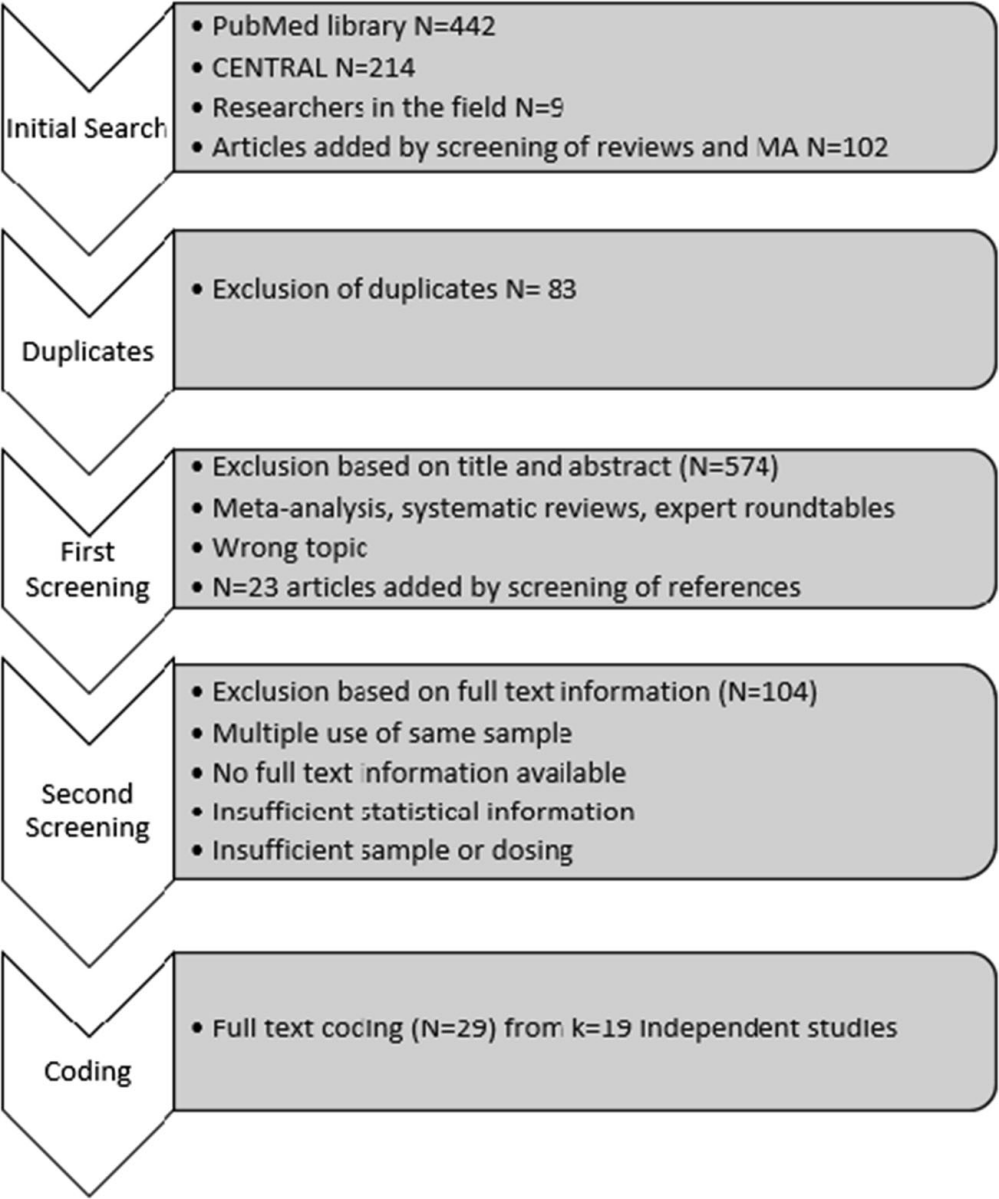
PRISMA search flow

The primary literature search returned a total of 767 references (Fig. 1). After exclusion of duplicates (*N* = 83) and the first screening on the titles and abstracts, 110 articles remained, to which we added another 23 by a manual search of reference lists. After full-text reading, 29 publications [28, 29, 31–59] describing 19 independent studies and including 27 independent samples fulfilled the inclusion criteria to be coded by two raters. Interrater reliability was calculated in order to show agreement between the two raters. Cohen’s kappa [60] yielded 85%, indicating a moderate to a high interrater agreement. Differences in data extraction were resolved by discussion. One study could be represented by multiple citations, if the supplemental articles added additional information on this study.

From the 29 reference studies, a total of 5629 patients from 27 independent samples with exudative AMD fulfilled the treatment protocol criteria and were included. The mean age was 76.8 (± 8.3) years, 56.2% were females. Five studies included multinational cohorts [28; 29; 31; 32, 33; 34; 35; *n* = 3351], while 3 were from Australia [36–38; 39; 40; *n* = 672] and 2 each from Norway [41–42; 43; *n* = 268], the USA [56–57; 58; *n* = 80], and Switzerland [44–45; 46; *n* = 465]. One sample each per country was added from Canada [47–49; *n* = 287], China [50; *n* = 85], Finland [59; *n* = 26], Japan [51–54; *n* = 247], and the UK [55; *n* = 148]. Further details regarding the single studies are displayed in Table 1.

### Treatment group comparison

In the overall sample, intra- and/or subretinal fluid were absent in 62.9% and 56.0% after 12 and 24 months, respectively. Treatment could be extended to ≥ 12-week intervals in 37.7% and 42.6% after 1 and 2 years of treatment, respectively. The retention rate of patients was overall high, yielding > 90% after year 1 and > 80% in year 2 indicating a high power of the data. After pooling of all data per drug, the groups differed regarding their potential to achieve a dry retina (absence of intra- and/or subretinal fluid) as an indicator of disease stability after 12 months (Ran = 56.3%, Afl = 64.5%, Bro = 71.5%) and after 24 months (Ran = 50.0%, Afl = 52.7%, Bro = 75.7%). Different numbers of patients were extended to ≥ 12 weeks for each of the three drugs after 12 months (Ran = 28.6%, Afl = 34.2%, Bro = 53.3%) and after 24 months (Ran = 34.2%, Afl = 47.7%, Bro = 41.7%; Table 2).

**Table 2:** Subgroup analysis for the absence of fluid, interval extension and retention rate

	12 months				24 months			
	*n*	*k*_S_	%	*p*	*n*	*k*_S_	%	*p*
Absence of fluid								
Ranibizumab 0.5 mg	1062	7	56.3	< 0.001	1081	5	50.0	< 0.001
Aflibercept 2.0 mg	442	5	64.5		1938	9	52.7	
Brolucizumab 6.0 mg	730	2	71.5		646	2	75.7	
Interval extension (12 weeks)								
Ranibizumab 0.5 mg	943	6	28.6	< 0.001	1339	7	34.2	< 0.001
Aflibercept 2.0 mg	770	7	34.2		2360	13	47.7	
Brolucizumab 6.0 mg	730	2	53.3		646	2	41.7	
Retention rate								
Ranibizumab 0.5 mg	943	6	90.5	0.69	1339	7	81.6	< 0.001
Aflibercept 2.0 mg	60	2	91.0		2266	12	81.7	
Brolucizumab 6.0 mg	730	2	91.6		646	2	88.6	

*k*_S_, number of samples included in the analysis

The *p* value represents the difference between all three groups

Based on a single data set and in the absence of real-life data confirming the trial findings, Bro seems to be superior to the two other drugs respecting absence of fluid as well as interval extension within the first year of treatment (*p* = < 0.001 each), whereas it remained superior only concerning the absence of fluid after 24 months (*p* = < 0.001; Table 2). The portion of eyes without intraretinal fluid declined in year 2 for Ran (from 56.3 to 50.0%) and Afl (from 64.5 to 52.7%), whereas it increased for Bro (from 71.5 to 75.7%). The opposite was observed for the portion of eyes with extended treatment to ≥ 12 weeks, which was increasing from 12 to 24 months (Ran from 28.6 to 34.2%, Afl from 34.2 to 47.7%), but decreasing for Bro (from 53.3 to 41.7%).

The overall pooled mean baseline visual acuity was 59.3 (SD = 14.0; CI = 57.7 to 60.9) ETDRS letters, while it increased to 67.6 (SD = 15.3; CI = 66.5 to 68.8) letters at 12 months and to 65.8 (SD = 18.4; CI = 63.8 to 67.7) letters at 24 months of follow-up. The pooled visual gain was 7.4 (SD = 12.6; CI = 6.4 to 8.4) letters at 12 months and 6.2 (SD = 14.7; CI = 5.6 to 6.9) letters at 24 months.

The pooled group of Ran and Afl reported a mean of 8.4 (SD = 1.9; CI = 8.1 to 8.8) injections during the first and 5.9 (SD = 2.2; CI = 5.1 to 6.7) injections during the second year whereas the number of injections was not reported separately for year 1 and 2 for Bro (Table 3).

**Table 3:** Subgroup analysis for visual acuity, visual gain and number of injections

	12 months									24 months								
						Between group-difference (*p*)									Between group-difference (*p*)			
	*n*	*k*_S_	Mean	SD	95% CI	Bro-Ran	Bro-Afl	Ran-Afl	Ran-Afl-Bro	*n*	*k*_S_	Mean	SD	95% CI	Bro-Ran	Bro-Afl	Ran-Afl	Ran-Afl-Bro
Visual acuity (VA)																		
Ranibizumab 0.5 mg	1415	8	67.9	11.5	66.1 to 69.7	0.99	0.59	0.64	0.86	568	4	67.7	18.5	65.1 to 70.3	n.a.	n.a.	0.11	n.a.
Aflibercept 2.0 mg	941	9	67.2	15.7	64.8 to 69.6					930	9	64.8	18.2	62.3 to 67.3				
Brolucizumab 6.0 mg	730	2	67.9	15.3	66.9 to 68.9					mv	mv	mv	mv	mv				
VA gain																		
Ranibizumab 0.5 mg	1443	8	7.6	12.7	5.6 to 9.6	0.43	0.44	0.86	0.61	1339	7	5.9	15.0	4.3 to 7.5	0.96	0.44	0.58	0.69
Aflibercept 2.0 mg	1151	11	7.4	12.3	6.0 to 8.8					2315	12	6.4	14.4	5.7 to 7.2				
Brolucizumab 6.0 mg	730	2	6.7	12.9	5.8 to 7.6					646	2	5.9	14.8	4.8 to 7.0				
Number of injections																		
Ranibizumab 0.5 mg	1415	9	9.0	2.3	8.6 to 9.4	n.a.	n.a.	< 0.001	n.a.	1037	4	7.4	2.6	5.1 to 9.8	n.a.	n.a.	0.06	n.a.
Aflibercept 2.0 mg	1101	10	7.9	1.3	7.5 to 8.3					1920	9	5.2	2.0	4.6 to 5.8				
Brolucizumab 6.0 mg	mv	mv	mv	mv^1^	mv					mv	mv	mv	mv	mv				

The *p* values represent the differences between all three groups

*mv*, missing value; *n.a.*. not applicable

^1^No information on the number of injections have been reported for brolucizumab (information could not be retrieved from the supplemental material either)

## Discussion

A realistic aim of treatment under anti-VEGF therapy is to maintain the functional gain and anatomic stability that was achieved by the end of the loading phase [61, 62]. Whereas only 25% of patients have maximal visual gain by the end of the lading phase, 14% of patients have gained substantially after that [63]. The fact that the portion of eyes for extension of the treatment to ≥ 12 weeks increased during the second year from 37.7 to 42.6% could indicate that disease stability increases over time. This possibility, however, is contradicted by the fact that the number of eyes without retinal fluid went back from 62.9 to 56.0%. Different factors could account for this finding, e.g., a more relaxed treatment extension strategy in year 2. This may, from a functional aspect, be well supportable in the short- and midterm, as demonstrated by the ALTAIR study [51, 52]. Another explanation might be patient adherence to treatment which was generally good but came down from 91.0 to 82.8%. However, the relatively good adherence probably can explain only a minor part of the changes over time. Under-treatment might thus be assumed. Indeed, the number of injections for Ran and Afl in the first year (9.0 and 7.9) compared to 7.4 and 5.2 during the second year indicates a robust reduction, but likely does not represent a relevant under-treatment and reflects clinical experience [46, 64]. This is also supported by a stable visual function between years one and two (Table 3). It seems that this reflects the challenges in balancing between under-treatment and a reduced treatment burden.

The calculation of the average treatment demand based on the number of injections per year or the mean treatment interval alone seems a rough estimate without taking into account the portion of eyes that changed from a dry situation at the end of year one to a non-dry one at the end of year 2. Disease stability may thus be a more robust marker to predict progression of the disease, but has not routinely been reported in recent series.

A general weakness of analyzing the treatment demand in studies where it is not the primary outcome may derive from the study design which does not reflect the last (possibly best) treatment interval but uses the pre-determined fixed annual time point. A robust calculation of treatment demand would have to include the last treatment extension interval without intra- and/or subretinal fluid before a given time point. It possibly should not be calculated after just 1 year under therapy. Nevertheless, evidence grows that the long-term (3–5 years) visual stability under a T&E strategy exceeds that of a PRN treatment with its inherent risk of under-treatment [65]. Though until recently only limited level 1 or 2 evidence existed in favor of a T&E strategy [66], this protocol rapidly found acceptance in many specialized retina centers [67].

Evidence regarding treatment extension beyond 12–14 weeks is scarce [58, 68]. The risk of severe vision loss, however, was reported to increase disproportionally with any further extension [69] which reflects the pathophysiological experience of the slow recovery of CNV perfusion after the disappearance of anti-VEGF effects [70–73]. It may thus require good arguments to exceed a treatment interval of 12–14 weeks for Ran and Afl. In contrast, the newer drugs in the pipeline such as faricimab, DARPins, and others as well as the recently approved Bro rise hope to exceed these experience-based maximal treatment intervals to 16 or more weeks in stable disease [74–77]. Conbercept, whose potential to extend the treatment interval to 12 or more weeks is currently assessed in a phase 3 trial (PANDA, ClinicalTrials.gov registration numbers NCT03577899 and NCT03630952) and seems to evoke a similar potency as Afl according to preliminary data (50). Considering that T&E protocols had been established systematically only a few years ago, it is not surprising that a manageable number of studies with different protocols provide limited information for treatment extension intervals, and the majority use the 12-month time point. The strength of this meta-analysis, on the other hand, is that it refers to almost 3000 eyes. Our data do not support the assumption that the portion of eyes reaching 12 weekly injection intervals will grow by up to 50% until 2 years of therapy, remaining widely stable after that regardless of the chosen anti-VEGF therapy [32]. If macular stability is achieved, this may be maintained in a subgroup of patients for up to 8 years [25, 78]. A baseline visual acuity of ≥ 70 ETRDS letters and an early satisfying functional response to treatment are more reliable predictors of long-term outcomes than lesion activity at the end of the loading phase [79] which may be outweighed by the presence of intra- and subretinal fluid as well as subretinal fibrosis at the end of the loading phase [80].

The first prospective clinical trial comparing a fixed monthly to a T&E regimen with Ran for nAMD, the TREX-AMD study, revealed that the T&E-treated eyes performed comparably to monthly treated ones; treatment interval could be extended to 11–12 weeks in 26% and ≥ 12 weeks in 18% of patients [56]. The 12-month outcomes were confirmed by a Canadian T&E study in which the treatment intervals in the T&E arm were extended to 12 weeks in 29.9% of Ran-treated eyes [50], and the TREND study [36]. Both were included in our meta-analysis, further supporting these findings, that more than 80% of eyes treated with Afl in 8 to 9-week intervals may become inactive within the first year if the treatment extension interval is not maximized [55, 81] and meets well with preclinical pharmacokinetic data [82, 83]. Not surprisingly, under a reduced treatment intensity after the first year, the rate of eyes without fluid went back to 71% after 2–3 years [55]. Its impact on changes in lesion size has as yet not been shown, but maybe a critical predictor of long-term functional stability.

Finally, it is important to carefully interpret the results. By summing up studies with unavoidable heterogeneity, the strength of conclusions may be limited. Nevertheless, they may provide interesting insight in a field where a direct comparison is not likely available as in the case of anti-VEGF therapies in exudative AMD. This also includes an imbalance in the sample sizes. Bro for example was only represented by two samples. Hence, namely the conclusions pertaining to Bro have to be drawn with the requested care.

In conclusion, disease stability as indicated by the absence of intra- and/or subretinal fluid is achieved in nearly two-thirds of eyes after 1 year, but declines under a T&E protocol to 56.0% by the end of the second year, indicating a possibly relevant long-term safety signal. This may reflect the challenges in balancing between under-treatment and a reduced treatment burden in the clinical situation.

## Data Availability

The data can be obtained upon request from the corresponding author.
